# Behavior-Based Video Summarization System for Dog Health and Welfare Monitoring

**DOI:** 10.3390/s23062892

**Published:** 2023-03-07

**Authors:** Othmane Atif, Jonguk Lee, Daihee Park, Yongwha Chung

**Affiliations:** 1 Department of Computer and Information Science, Korea University, Sejong City 30019, Republic of Korea; 2 Department of Computer Convergence Software, Sejong Campus, Korea University, Sejong City 30019, Republic of Korea

**Keywords:** video summarization, dog behavior recognition, computer vision, video monitoring system, dog health and welfare, visualization

## Abstract

The popularity of dogs has been increasing owing to factors such as the physical and mental health benefits associated with raising them. While owners care about their dogs’ health and welfare, it is difficult for them to assess these, and frequent veterinary checkups represent a growing financial burden. In this study, we propose a behavior-based video summarization and visualization system for monitoring a dog’s behavioral patterns to help assess its health and welfare. The system proceeds in four modules: (1) a video data collection and preprocessing module; (2) an object detection-based module for retrieving image sequences where the dog is alone and cropping them to reduce background noise; (3) a dog behavior recognition module using two-stream EfficientNetV2 to extract appearance and motion features from the cropped images and their respective optical flow, followed by a long short-term memory (LSTM) model to recognize the dog’s behaviors; and (4) a summarization and visualization module to provide effective visual summaries of the dog’s location and behavior information to help assess and understand its health and welfare. The experimental results show that the system achieved an average F1 score of 0.955 for behavior recognition, with an execution time allowing real-time processing, while the summarization and visualization results demonstrate how the system can help owners assess and understand their dog’s health and welfare.

## 1. Introduction

At the beginning of the 20th century, structural changes in South Korea’s society, such as the spread of individualism, the rise in divorce rates, and the decline in fertility rates, contributed to the aging of the population and a significant increase in single-person households [1]. For people living alone, to cope with loneliness, many opted for raising pets to serve as an alternative to family members and help them with stress [2,3]. This led to an increase in the number of households with pets from 3.59 million in 2012 to 6.04 million in 2020, which represents 30% of the country’s households [4,5].

Among the pets commonly owned, dogs are a popular choice because of the benefits associated with raising them. They motivate to engage in activities, such as walking and exercising together, helping owners sustain their physical [6,7,8] and mental health [9,10,11]. In fact, dogs are currently leading the pet market in South Korea, with a total of approximately 5.2 million raised in 2021 and a presence in 80% of households with pets [4,12]. However, while the number of dogs raised remained relatively stable between 2019 and 2020 [13], data from the Korea Data Exchange (DEX) showed a 60% increase in veterinary hospital visits during the same period [14]. While this shows that owners care for their dogs’ health and are spending money and time to maintain it [4], the increasing veterinary costs have reached an average of $72 per visit, which represents a burden for more than 82% of owners [15]. This financial burden increases when owners take their dogs for random checkups in the hope of discovering and treating health issues. For these reasons, owners need to better understand the health (physical and mental) and welfare of their dogs so as to limit veterinary visits to the necessary ones, rather than performing frequent checkups.

Although medical conditions of some dogs require tests and expert opinions for diagnosis, research has shown that there exists a strong bond between a dog’s welfare and health and its behavior [16,17,18,19]. In fact, not only can monitoring a dog’s behavior help the owner understand their pet’s health state and improve the accuracy of the expert’s diagnosis, but in some cases, behavior is the only clinical sign that experts can rely on [20,21,22]. Thus, monitoring dogs’ behavioral information is necessary because—when accurately collected—such data can help the owner discover abnormalities and provide useful insight to the expert in making an accurate diagnosis. Consequently, this would help the expert start the treatment early, improving the chances of a quick recovery and reducing veterinary visits, which would relieve some of their financial burden.

Although owners generally tend to take notice of their dogs’ behaviors, it is difficult for them to do so constantly, especially since it is common for dogs to be left unattended. For example, in South Korea, dogs spend an average of five to seven hours alone at home [4], a time during which owners cannot directly observe their behaviors [23]. Hence, owners are likely to miss key behavioral signs and pattern changes that can help assess their dogs’ states, especially because some signs are displayed mainly when the dog perceives its owner’s absence [23,24,25]. In fact, dogs with physical and separation-related health issues when left alone tend to display restlessness, increased activity, pacing, and some undesirable behaviors, such as excessive vocalization, unlike healthy dogs, which are likely to mainly show long periods of passive behavior and locomotion [26]. Owing to that, and since the owner’s presence can cause the dog to synchronize its behavior with theirs [27], keep it attentive to the owner and trigger interactions, such as playing, which can affect the dog’s behavior [28], it is important to observe the dog’s behavior when alone and keep track of them [29]. This can provide more consistent information about its behavioral changes. Therefore, in this paper, we propose a method to monitor a dog in an illuminated indoor environment, recognize its behavior, and provide an effective way to visualize it to help owners and experts better understand their health and welfare.

After reviewing previous studies on dog behavior recognition, we identified related studies [30,31,32,33,34,35,36,37], which are summarized in Table 1.

Different methods have been introduced to recognize dog behavior. In this context, dog behavior refers to static (e.g., sitting and sleeping) and active behaviors (e.g., walking, barking, and jumping). Early methods used statistical classification [30], DTW distance [31], and discriminant analysis [32] to classify selected dog behaviors, providing proof of concept. Later, with the improvement in machine learning techniques and the increase in their popularity in different fields of research [36,38,39,40], more methods have been employed to recognize dog behaviors. For instance, Chambers et al. [34] aimed to validate dog behavior recognition, with a focus on eating and drinking, using FilterNet and a large crowd-sourced dataset. Using their method, Aich et al. [33] trained an artificial neural network (ANN) on sensor data to detect dog behaviors and classified their emotional states (positive, neutral, or negative) based on their tail movements. In addition, Wang et al. [36] classified dogs’ head and body postures separately using long short-term memory (LSTM) on sensor data, and then used complex event processing (CEP) to better differentiate between behaviors with similar body postures. Kim et al. [37] used a convolutional neural network (CNN) with LSTM on fused image and wearable sensors data to compensate for the shortcomings of this latter caused by noise in a multimodal system to improve the classification of a dog’s behaviors. By contrast, Fux et al. [35] used object detection to track a dog inside a clinical setting, extracted features from its movements, such as straightness, and classified them using random forest to recognize ADHD-like behaviors.

While these methods achieved good results, their main purpose was to confirm the possibility of recognizing dog behaviors: they focused mainly on and concluded their work on dog behavior classification. However, with only that as a result, understanding a dog’s health and welfare remains a challenging task for owners, because relying solely on the recognized behaviors and manually reading through them to try to gain insight is impractical, time-consuming and may result in missing important details. Instead, in this paper, we propose a method that provides owners with summarization and visualization of their dog’s recognized behaviors to help them effectively visualize them and perceive important patterns and behavioral changes that would help assess its health and welfare. Furthermore, for better assessment, it is required at times to retrieve and visualize some behaviors to examine their intensity and significance [41,42], which makes it necessary for this method to collect video data as input. Most studies did not consider extracting the dog’s location information, which can be useful in detecting movement patterns [35]. In addition, only a few studies have considered real-time processing, which is needed to ensure quick feedback in cases of an alarming increase in the frequency of poor welfare-related behaviors that require immediate attention [41,42]. Finally, because the owner’s presence can influence the dog, to ensure a consistent observation of behaviors, including a preprocessing step to select only the data where the dog is alone without any person present can provide an additional advantage. This further supports the need for using image data in our system as it facilitates the detection of the presence of subjects of interest (i.e., dogs and people) in the input data. Hence, in this study, to overcome the limitations of previous studies, we used a camera-based system to monitor, recognize, and provide summarization and visualization of a dog’s location and behaviors when left unattended to help effectively understand its health and welfare. Accordingly, the system includes the following features.

1. Handling video data containing dogs and people, matching real-life situations, and automatically detecting and selecting the data where the dog is alone.
2. Tracking the dog’s location and performing behavior recognition in real time to provide live visualization and urgent alerts to owners.
3. Summarizing the dog’s video using its tracked location data and recognized behaviors and providing an effective visualization that helps draw insightful information to understand and assess its health and welfare.

We propose an end-to-end system for dog video summarization based on detected behaviors and location information. The system uses the object detection model You Only Learn One Representation (YOLOR) to detect the dog’s location, followed by an adapted sequence bounding box matching algorithm to correct missed detections before selecting only sequences of images where the dog is alone. Subsequently, the images are cropped to focus on the dog and a two-stream CNN-LSTM is used for behavior recognition based on RGB and optical flow images. Both outputs from YOLOR and CNN-LSTM are stored as log data and then used to generate graphs to provide effective and efficient visualization summaries for owners and dog experts to assess the dog’s physical and mental health and welfare.

## 2. Related Work

The most important task in our system is the summarization of the dog’s video data based on its recognized behaviors to provide a visualization of the results using analytical tools. Thus, to select a suitable approach for this task, we first explored the existing video summarization methods.

Video summarization is a research field that aims to create a concise summary of a video by selecting the most important parts. It is generally divided into scene-based, where visual features are extracted to select key frames [43], and content-based [44], which uses information related to the content and semantics in the video, such as objects [45] and actions [46,47]. In terms of action-based summarization, although close to our purpose, the proposed methods mainly targeted humans and relied on motion [46,48], making them unsuitable for our system because we targeted dogs’ behaviors, which include not only actions (active behaviors) but also static behaviors that lack motion. However, because this type of summarization is performed in two steps, action recognition and summarization, by following a similar approach and using a behavior recognition method as a first step, we can achieve our summarization purpose. Accordingly, it is important to select an approach that delivers accurate recognition of dog behavior for our system to ensure good summarization performance. To this end, we examined recent methods used for animal behavior recognition to select a suitable method for dog behaviors and found that models based on the CNN-LSTM approach have been widely used for their performance. For instance, Chen et al. [49] used VGG16-LSTM to recognize aggressive behaviors of pigs in pig farms for injury prevention, and Chen et al. [50] used ResNet50-LSTM to classify the drinking behaviors of pigs to verify their adequate water intake. Recently, EfficientNet-BiFPN-LSTM was proposed by Yin et al. [51] to recognize the motion behavior of a single cow to help with farm monitoring. Each of these methods showed good results in terms of behavior recognition, proving that the method is suitable for our goal. However, because the background and presence of multiple animals make it difficult to extract optical flows, these methods rely on a single-stream CNN-LSTM, using only RGB frames. Because the use of both RGB for appearance and optical flow for motion can lead to better recognition performance [52], guaranteeing by that a more accurate summarization, especially in our system, which targets both active and static behaviors, a two-stream CNN-LSTM model is more appropriate for us to use.

Accordingly, we propose an end-to-end system for behavior-based dog video summarization using four modules. The first module collects and preprocesses the image sequences and forwards them to the second module, namely, the YOLOR-based dog-alone sequence retrieval module. At this level, object detection is performed using YOLOR-P6 [53] to detect dogs and people, followed by sequence bounding box matching [54] to correct missed detections. The YOLOR-P6 model was used because it provides one of the best speed and accuracy tradeoffs among the object detection models. Further processing is performed to select sequences of frames containing a dog alone, and their detection results are saved as a dog’s location log data. After that, the selected frames are spatially cropped with a focus on the dog to reduce background noise and location dependence. The sequences of cropped images are then forwarded to the dog behavior recognition module, where a two-stream EfficientNetV2B0 is used to extract features from the RGB and optical flow frames, generated using the global motion aggregation (GMA) algorithm [55] and then a bidirectional LSTM to extract temporal features from the cropped dog sequences. The specific models and algorithms used in this module were selected because they guarantee good performance and inference speed. Finally, the detected behaviors are saved as log data and forwarded to the dog-behavior-based video summarization and visualization module. At this stage, data visualization techniques are utilized to generate an effective visual summarization of the dog’s location and behavior through different graphs to help identify patterns and features that can allow a better understanding and assessment of the dog’s health and welfare.

## 3. Behavior-Based Dog Video Summarization System

**Figure 1:**
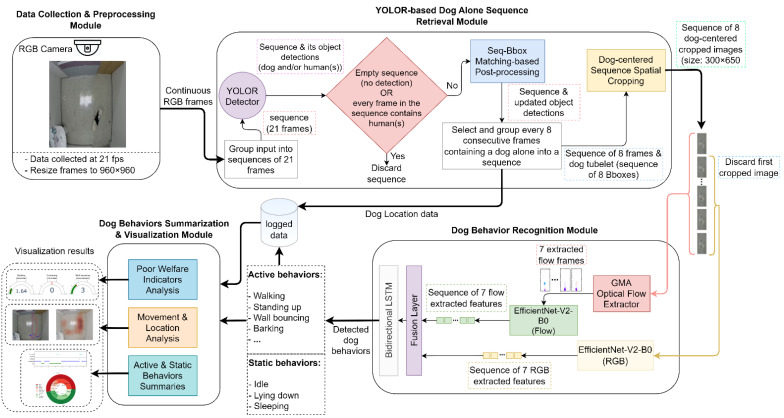
Overall architecture of the proposed behavior-based dog video summarization system.

The architecture of the proposed behavior-based dog video summarization system is illustrated in Figure 1.

### 3.1. Data Collection and Preprocessing Module

The image data used by the system were transmitted at a frame rate of 21 fps from a top-down angle RGB camera installed in the ceiling to minimize occlusions and overlapping with people and other objects. This angle also provides an intuitive way of localizing the objects of interest inside a room and studying their movements, making it commonly used in monitoring systems [49,50,51,52,56]. In this module, the collected RGB images are resized to 960×960. pixels to match the input size of the YOLOR model and are then forwarded to the next module.

### 3.2. YOLOR-Based Dog-Alone Sequence Retrieval Module

The data received at this stage contain frames with a dog alone, frames with one or more people, frames with people and a dog, or frames of an empty room. To ensure consistent observations and prevent the owner’s presence from influencing the dog’s behavior [27], this module selects only sequences of frames where the dog is alone and discards the remaining ones. YOLOR object detection was first used to detect humans and dogs in each sequence. If a sequence contains no detections or contains at least one human detection in every image, it is discarded. Otherwise, the sequence bounding box (Seq-Bbox) matching algorithm is applied to handle missed detections. Subsequently, the image sequences containing a dog alone were retrieved, and their corresponding bounding boxes were saved as dog location log data. Finally, an algorithm was applied to crop the images in each sequence to focus on the dog using a unified padded bounding box, generated from the dog’s bounding boxes. In short, this module receives continuous RGB frames as the input and output sequences of eight dog-focused cropped images. The entire process is described in detail in the following subsections.

#### 3.2.1. Dog and Human Object Detection

Owing to recent advances in deep learning, the field of object detection has shown continuous improvements in speed and performance and led to the development of methods and models targeting several areas of research and covering different scenarios [57,58]. Among the existing object detection models, You Only Look Once (YOLO) [59] and its variants have attracted considerable attention because of their performance and real-time inference speed, making them a popular choice for monitoring systems [37,56,60,61].

In this module, the YOLOR model was used for object detection of dogs and humans. YOLOR is a recent YOLO variant with a network that integrates both explicit and implicit knowledge to learn one general representation. More specifically, the YOLOR-P6 subvariant, which offers one of the best performance and speed tradeoffs guaranteeing real-time inference, was used for human and dog detection on each frame received and output the class, score, and bounding boxes of every object detected. First, the frames received from the previous module were grouped into sequences of 21 consecutive frames for object detection and analysis of each sequence separately. The sequence length was set to 21 frames to match the camera’s frame rate. After object detection is performed on the images in a sequence, if there is no detection in any frame of the sequence, or if at least one human is detected in every frame of the sequence, the whole sequence is discarded and the module moves to process the next sequence. Otherwise, the sequence and its detection results are retained for further analysis to retrieve from it sequences of data containing a dog alone. However, because YOLOR is a per-frame detection model, when performing object detection on a sequence of frames such as in this case, missed detections can occur due to factors such as unusual object poses [62]. To ensure the correct retrieval of frame sequences with a dog alone, it is important to handle such missed detections. In general, when the detection model targets a single object, methods such as linear interpolation can be used directly to replace the missing detections based on the detections in the closest surrounding frames [37]. However, as we target multiple objects (a dog and humans), in some scenarios when there is a missed detection, nearby frames can contain multiple detections. In such a case, to select the exact detections that need to be used from nearby frames to generate the missed detection, we first need to identify and match in a sequence all the detections that correspond to each object. For this purpose, the Seq-Bbox matching postprocessing method proposed by Belhassen et al. [54], which specifically performs this matching, was used, as explained in the next section. Accordingly, the sequences of the 21 frames that were not discarded are forwarded with their corresponding detection results to the Seq-Bbox matching postprocessing unit to handle missed detections.

#### 3.2.2. Seq-Bbox Matching-Based Postprocessing

This unit of the module takes a sequence of 21 frames and its detections as input, uses Seq-Bbox matching to correct any missed detections, and then outputs sequences of eight frames containing a dog alone and their detection results. The Seq-Bbox matching postprocessing algorithm used here is the one proposed in [54], and it uses the Seq-Bbox matching technique to match detections every two consecutive frames based on the distance score shown in Equation (1).

(1) $$distance=\frac{1}{similarity}=\frac{1}{IoU\times \left(Vctr_{i}\cdot Vctr_{j}\right)}\;$$

where intersection over union (*IoU*) represents the geographical proximity of two bounding boxes and the dot product of the two classification score vectors depicts the similarity in the semantics of bounding boxes *i* and *j*.

**Figure 2:**
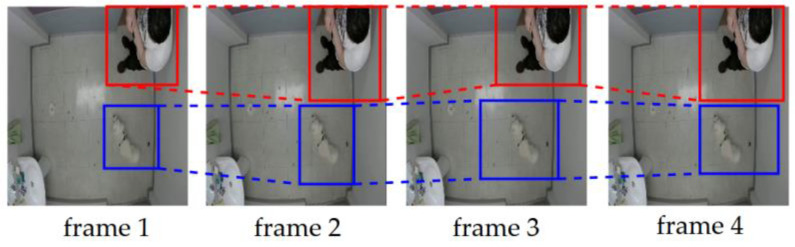
Examples of tubelets. The tubelet in red represents the human’s bounding boxes and the tubelet in blue represents the dog’s bounding boxes.

Through the matching of detections in a sequence, tubelets that represent sequences of bounding boxes specific to objects detected in a sequence are generated. Figure 2 shows examples of a tubelet of a human marked in red and a tubelet of a dog marked in blue.

**Figure 3:**
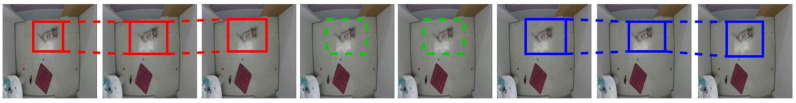
Example of linking two tubelets, Ti in red and Tj in blue, and generating missed detections (green dotted boxes).

After generating tubelets and to generate missed detections, the bounding boxes of the last frame belonging to each tubelet Ti are matched with the bounding boxes of the first frame of each tubelet Tj, given that the first frame of Tj starts temporally later than the last frame of Ti. Once the two tubelets are linked, the missing boxes in between are generated using bilinear interpolation. Figure 3 shows an example of tubelets linking a tubelet Ti (in red) and Tj (in blue) and the missed detections generated through bilinear interpolation (in green).

Furthermore, to prevent matching tubelets of different objects, a threshold k is used to limit the accepted temporal interval between the candidate tubelets Ti and Tj and only link tubelets if the number of frames between the last frame of Ti and the first frame of Tj is less than k. Following the experimental method used in [54] to select a value for k, the optimal value for k on our dataset was set to k=15.

Through Seq-Bbox matching postprocessing, missed detections are reduced in each sequence of 21 frames, and for each object, a tubelet is generated. Subsequently, tubelet matching and linking are applied between every current and previous sequence $S_{n}$ and $S_{n-1}$ to generate any missed detections in between. Once the final tubelets are generated, they are analyzed to select the sequences of frames containing dog tubelets that do not overlap with human tubelets, as these represent sequences of images with a dog alone, and then the sequences are regrouped in sequences of eight frames. Unlike in the previous unit, where sequences contained 21 frames, at this stage, the grouping of frames with a dog alone uses eight frames as sequence length to match the input size of the EfficientNet-LSTM. Subsequently, new sequences of length 8 are forwarded to the next unit with their corresponding dog tubelets. Meanwhile, the dog’s bounding boxes from each frame, which represent the dog’s location tracking, are saved as log data.

#### 3.2.3. Dog-Centered Sequence Spatial Cropping

At this point, and to improve the behavior recognition results, the images in each sequence are spatially cropped to put more focus on the dog. This helps concentrate learning on our specific target region to explore its contextual features, reduce the impact of less significant elements, and prevent the model from associating actions with specific locations [52,63]. Accordingly, this unit receives sequences of eight frames with its dog tubelets and since only spatial cropping is performed at this level, the sequence length is maintained, and hence the output is a sequence of eight spatially cropped images.

Because the dog’s tubelets are already detected through the YOLOR model and Seq-Bbox matching, it is possible to use them for image cropping with a focus on the dog. Nevertheless, because every bounding box in the tubelet has a different size, directly using them to crop the dog in every frame of the sequence will result in a sequence of cropped images of different heights and weights. When resized to match the image input size of the CNN-LSTM model, the proportions of the dog will differ from one frame to the next in the sequence, which will negatively affect the optical flow and image feature extraction. Thus, for cropping while maintaining the dog’s proportions, we propose a simple algorithm to unify the bounding boxes across each tubelet and use the unified bounding box to crop every frame in the sequence.

**Figure 4:**
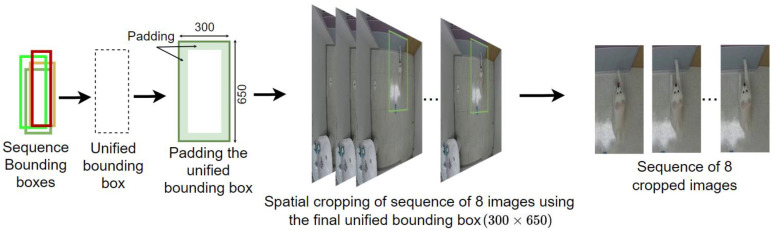
Process of generating and utilizing a unified padded bounding box for spatial cropping on a sequence. In this scenario, padding is applied equally on each side of the box.

The algorithm proceeds by examining the $x_{min}$, $x_{max}$, $y_{min}$ and $y_{max}$ values of every bounding box in a sequence of eight frames, selecting the smallest values of $x_{min}$ and $y_{min}$ and the highest values of $x_{max}$ and $y_{max}$. These values are used to define a unified bounding box that includes all areas covered by the bounding boxes in a sequence. Furthermore, to ensure that all cropped sequences have a similar size, the dataset was analyzed to approximate the largest possible height and width of a unified bounding box, which was found to be 300×650, and padding was used on the unified box to reach that size. The algorithm first attempts to add padding equally from each side of the bounding box vertically and horizontally; however, when the padding exceeds the limit of the total image size on one side, excess is added to the other side. Finally, the unified and padded bounding box was used to crop every image in the sequence to produce a sequence of eight dog-centered cropped images (dog-centered tubelet). The process of generating, padding, and using a unified bounding box to spatially crop images in a sequence is illustrated in Figure 4.

The cropped images were then resized to 224×224 to match the input size of the CNN-LSTM before being forwarded to the behavior recognition module.

### 3.3. Dog Behavior Recognition Module

In this module, we used a CNN-LSTM model for dog behavior recognition, as it has been demonstrated to be efficient and perform well, making it popular for use in animal behavior recognition [49,50,51]. In particular, as shown in the comparative analysis presented in the work done by Yin et al. [51], when used as a spatial feature extractor, the EfficientNet [64] model was able to outperform other commonly used CNN models such as VGG16 and ResNet50 and matched the results of DenseNet169 in behavior recognition while using significantly fewer parameters. For this reason, we adopted a similar approach in our system, but instead of the EfficientNetV1, we used EfficientNetV2, which was introduced by Tan et al. [65] to solve some of the bottlenecks of its predecessor. This was accomplished through a combination of scaling and training-aware neural architecture search (NAS), with the extensive use of MBConv and fused MBConv in early layers to increase both training speed and parameter efficiency [52,64,65]. This makes it a suitable choice for our architecture because it can guarantee real-time application through fast inference. More specifically, the B0 variant was selected because it had the lowest number of parameters. Because using both RGB and optical flow can lead to improved results [52,66], in this module, we use a two-stream CNN-LSTM to recognize dog behaviors, using EfficientNetV2 and a bidirectional LSTM (Bi-LSTM). The Bi-LSTM was selected because of its good performance with time-series data and capability to integrate future and past information when predicting, which allows it to better define time boundaries for actions [52].

Accordingly, the sequence of eight cropped images received in this module was used for dog behavior recognition through a two-stream EfficientNetV2B0-Bi-LSTM model. The sequence was first input to the GMA [55] algorithm to extract the optical flow between every two consecutive RGB cropped images. Consequently, each sequence of eight cropped images is used by GMA to extract flow and generate a sequence of seven optical flow images. The GMA algorithm was used because it provides one of the lowest end-point error (EPE) values on the Sintel dataset benchmark [67] while ensuring low latency. Subsequently, due to the mismatch between the sequence of seven flow frames and the sequence of eight cropped RGB images, the first cropped RGB frame is dropped to allow both streams of data (RGB and flow) to have the same sequence length of seven frames, which will later guarantee that the LSTM layer receives a sequence with time steps of consistent features vector size. At this stage, each sequence is fed to its respective EfficientNetV2 model that was pretrained on similar data to serve as a spatial feature extractor to learn appearance features from the RGB sequence and motion features from the optical flow sequence. For use as a feature extractor, the last dense layer of both EfficientNetV2 models was removed to output a feature vector of size 1280 for each sequence of RGB and flow. Subsequently, both sequences are fed to a fusion layer, to be concatenated before being fed to the Bi-LSTM as one sequence of seven fused feature vectors, each of size 2560. At the Bi-LSTM level, the temporal features in the sequence were extracted and a softmax layer used to classify them and recognize the dog’s behavior, which was then saved in the database as log data and simultaneously forwarded to the next module. The behaviors recognized in this module are described in more detail in Section 4.1.

### 3.4. Dog Behavior Summarization and Visualization Module

At this stage, the dog’s detected location and behavior are simultaneously received by this module to provide real-time feedback and saved as log data in the database. Simple postprocessing is applied by grouping the detected behaviors to define their occurrence, length, and temporal boundaries. In addition, in this module, data visualization techniques were used to convert the saved location information and summarize the behaviors from the log data into an effective visual representation. By doing so, the system can help owners and experts identify behavioral features, anomalies, and patterns to better understand the dog’s health and welfare.

The following subsections present the different types of graphical and visual summarization used to cover different aspects of dog behavior that can help assess their state.

#### 3.4.1. Poor-Welfare Indicator Monitoring

Ensuring that a dog benefits from good welfare is one of the main responsibilities of an owner, which is why it is important for them to understand when their dogs are showing signs of possible poor welfare, and to manage and improve it. While precisely recognizing this is a difficult task, previous research has defined some dogs’ behaviors as possible indicators of poor welfare when they are displayed in high frequencies. Accordingly, we selected potentially abnormal behaviors displayed by the dogs in our data collection experiments to demonstrate how they can be used to detect possible poor welfare. Table 2 shows these behaviors and the frequency or time threshold starting from where the behavior becomes significant for poor welfare [68].

These behaviors can potentially cause physical harm to the dog and disturbance to the community and may require quick intervention. Therefore, providing a visualization of their level of occurrence in real time represents an effective way for owners to monitor them. For this purpose, we employ gauge charts to display the measure of occurrence for each of those behaviors to visualize when they reach their respective thresholds, and owners can choose to be notified when that happens.

#### 3.4.2. Dog Movement Visual Summary

A dog’s movement and locomotion can provide a basis for assessing aspects of its health and welfare. In fact, factors such as ambulation patterns and restlessness levels have been associated with health and welfare issues such as physical pain, stress, and hyperactivity-related disorders [22,69,70,71]. In addition, some movement patterns, such as pacing and circling, are considered signs of anxiety in dogs [72,73]. Therefore, our system provides a visual summary of the dog’s spatial movement based on the logged location information to allow owners and dog experts to observe and look for similar signs.

The dog’s movement visual summary is presented in two graphs. The first is a heatmap generated using the saved bounding boxes of the dog to represent the areas covered by the dog’s movement to better understand the dog’s movements and activity level, whereas the second represents the trajectory of the dog’s movement. This second graph uses the centroid of the bounding boxes from the log data and connects them to each other using a color map in order to define the timeline of the trajectory. In addition, circles were used to highlight the beginning and end points of a dog’s movement.

#### 3.4.3. Summarization of Dog’s Displayed Behaviors

Two graphs were used to summarize and visualize the displayed behaviors of the dogs. The first is a nested doughnut chart that displays the percentage of active and static behaviors alongside the specific behaviors belonging to each category, which provides a general understanding of the dog’s displayed behaviors as a whole and allows observation of changes in the behaviors displayed. This is important, as increases or decreases in the frequencies at which some behaviors occur are a common sign of health and welfare issues [21,22,74]. A scatterplot was employed as a timeline to visualize the dogs’ behaviors and their time duration. Based on this, patterns such as the phases of resting and sleeping, which are important welfare indicators [75], can be observed, in addition to the levels and types of activity displayed.

## 4. Experimental Results

### 4.1. Data Collection and Datasets

**Figure 5:**
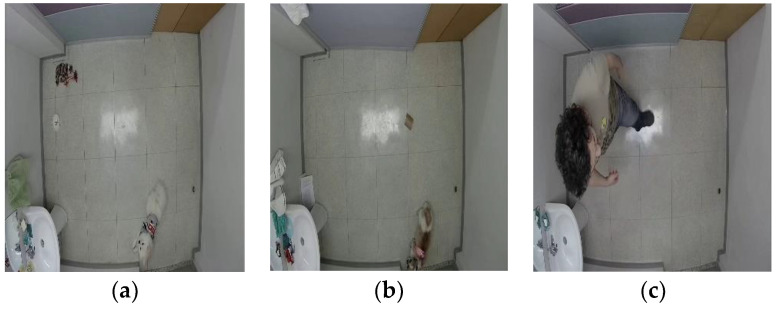
Examples of image data collected: (**a**) example of data collected with a spitz; (**b**) example of data collected with a shih tzu; (**c**) example of data collected with a human.

The data were collected in a laboratory with a CCTV camera (Hikvision DS-2CE56D0T-IRMM (2.8MM), Hangzhou, China) recording frames with a resolution of 960×1080 sampled at 21 fps, mounted on the ceiling at a direct top-down angle with the participation of two small dogs: an 8-year-old rescue shih tzu and a 2-year-old spitz. The experiments’ recordings were conducted in a room located in Sejong City, South Korea, with the consent and presence of the owners and under continuous direct or remote supervision when the dog was left alone inside the recording area. The first few visits were done to ensure that the dogs had time to discover and familiarize themselves with the environment to avoid causing them any stress when collecting data. In addition, the recordings were limited to a maximum of 20 to 30 min with breaks when needed to prevent any discomfort, water was provided along with the dog’s own toys and items used at home, and the room temperature was maintained at a comfortable level. The camera recordings started before the arrival of the dog, during the breaks, and after the experiments were conducted to collect diverse data, including frames of the empty room, humans with and without the dog to be used for the object detection model training. The setup of the room was changed slightly to produce a different background, and people were invited to the recording area on different occasions for daily life tasks for the purpose of obtaining more real-life data. Figure 5 shows examples of the collected data.

The image data of both humans and dogs were labeled with bounding boxes for object detection training, and only the dog was labeled for behavior recognition in the data where it was alone. The dataset used for object detection contained 12,000 samples from the shih tzu, 16,000 samples from the spitz, and 15,000 samples from different people. In order to select the length of the images sequence used as input to the EfficientNet-LSTM model, we considered the findings of the analysis done by Zhang et al. [66], where they compared results of a two-stream approach with different input sequence lengths on various datasets. Consequently, we determined that a sequence of length eight frames, which generally leads to good results, would work for our model, and this was further validated through our experimental results. Table 3 shows the number of sequences of eight images used for the behavior recognition training for each of the behaviors displayed by the dog in the data collection experiments alongside their descriptions. External stimuli were limited to avoid any bias and to collect data on the dogs’ naturally displayed behaviors. Because some of the dogs’ collected behaviors were less frequent than others, the samples of the frequent ones were limited to reduce the dataset imbalance while selecting random samples from different experiments.

### 4.2. Experimental Environment and Setup

The system implementation and the experiments were all conducted using Python 3.8 in an Anaconda environment on a computer running Windows 10 with an Intel i7 8700 K CPU, 32 GB of RAM, and an RTX 2080Ti GPU. The YOLOR object detection model was trained using the PyTorch library following the officially recommended environment, whereas the EfficientNetV2 and LSTM models were trained with the TensorFlow 2.8 library. The GMA code was based on the official implementation. Both object detection and behavior labeling were performed using the ViTBAT [76] software, and a Python script was implemented to convert the bounding box labels to the YOLO format. It is also worth mentioning that the YOLOR-P6 model was used to automatically generate object detection labels after it was trained on part of the data that were manually labeled to speed up the labeling process on the remaining data. The tool introduced in [77] was used to calculate the mean average precision (mAP) for object detection evaluation. The visualization tools were implemented using Matplotlib and Plotly libraries.

### 4.3. Performance Evaluation

#### 4.3.1. Evaluation Metrics

The following Equations (2)–(5) represent the different metrics used for the evaluation of the models in the second and third modules of our proposed system.

(2) $$mAP=\frac{\sum_{1}^{C}AP\left(C\right)}{C}$$

(3) $$Precision=\frac{TP}{TP+FP}\;$$

(4) $$Recall=\frac{TP}{TP+FN}\;$$

(5) $$F1\;score=\frac{2\times Precision\times Recall}{Precision+Recall}\;$$

where *mAP* is the mean average precision, *AP* is the average precision, *C* is the number of classes, true positive (*TP*) denotes the dog’s behaviors that are correctly classified as true, false positive (*FP*) is the number of falsely identified dog behaviors, and false negative (*FN*) is the number of behaviors incorrectly classified as false.

#### 4.3.2. YOLOR-P6 Dog-Alone Sequence Retrieval Results

The data used for the YOLOR-P6 object detection model training included samples of labeled images of dogs and people and images of an empty room. The dataset was divided according to a ratio of 8:2, resulting in training samples with 34,400 images and the validation samples with 8600 images. YOLOR-P6 was trained for 300 epochs with an input size of 960×960 pixels using default hyperparameters. To evaluate and compare YOLOR-P6 object detection with and without the seq-Bbox matching algorithm to confirm the effectiveness of the latter, the mean average precision (mAP) and latency in milliseconds were calculated, and the results are presented in Table 4.

As seen in Table 4, the seq-Bbox matching postprocessing improves the results of the YOLOR-P6 model, especially the AP of the dog which reached 0.962, while only requiring 0.19 ms in additional latency.

Furthermore, precision, recall, and F1 score were used to evaluate the effectiveness of the dog-alone retrieval module and confirm that it can accurately retrieve sequences of data containing a dog alone. We used a different set of sequences of eight images containing four different scenarios: a dog alone, one or more people, a dog with one or more people, and images of an empty room. The results, as seen in Table 5, confirm the efficacy of this method in differentiating between the different types of sequences and hence its efficacy in retrieving the sequences of data where the dog is alone.

Although the average precision (AP) for people detection was relatively low, the results of the retrieval were significantly better, and this is due to the fact that some of the scenarios used in both evaluations (Table 4 and Table 5) contained multiple people, and in the most challenging ones, some of them stood too close to each other, which affected the object detection results. However, for the same scenarios, the detection of at least one person allows the sequence retrieval module to correctly classify them, especially in the dog and person scenarios, which explains the good retrieval results. Based on these results, we can confirm that this module performs well and provides accurate tracking of a dog’s location information and retrieval of sequences of a dog alone.

#### 4.3.3. Dog Behavior Recognition Results

In this section, we present the results of two experiments. The first is used to validate the effectiveness of the EfficientNetV2-LSTM model proposed in this paper using Precision, Recall and F1 score, and the second is to compare the results of the behavior recognition, with other models used for action-based summarization and behavior recognition. The data used in both the experiments are listed in Table 3. To train the models, the dataset was divided into training, validation, and testing data at a ratio of 7:2:1. EfficientNetV2 used for RGB images was trained for 60 epochs, whereas the one used for optical flow images was trained for 150 epochs, both of which were trained using the Adam optimizer with a learning rate of 0.00005 and an input size of 224×224. In contrast, the Bi-LSTM network consists of one layer of 60 hidden units, uses a dropout rate of 0.5, and 0.1 recurrent dropout, and it was trained for 200 epochs using the Adam optimizer with a learning rate of 0.0005 and categorical cross-entropy as a loss function. A softmax activation layer was used on top of the LSTM to classify the results and recognize the behaviors.

The results of the first experiment show that our proposed method achieved an average F1 score of 0.955, as shown in Table 6, confirming the performance of the proposed method. As seen from the results, such behaviors as “idle” have relatively lower accuracy, as it is sometimes hard to differentiate it from other behaviors with similar postures, such as “lying down” or “walking”, when the dog’s walking speed is low. Similarly, “standing up” and “wall bouncing” sometimes share similar features in posture and motion, which can lead to misclassification.

In the second experiment, other recent methods for action and behavior recognition were used to compare their recognition results with our proposed method. The TDMap–CNN method by Elharrouss et al. [46] used in their proposed action-based summarization method, VGG16-LSTM [49] and ResNet50-LSTM [50] used for animal behavior recognition, and our proposed EfficientNet-LSTM, using full-size images and cropped images as input data. The TDMap-CNN [46] was originally designed to generate a background using cosine similarity and to employ the generated background for segmentation and track people. However, in our case, as there were instances where the dog remained inactive for long periods, the generated background ended up including the dog and affected segmentation. To address this issue, for each video used, we manually selected a portion in which the dog was in constant motion to generate the background and use it. On the other hand, the second and third models were trained as single-stream CNN-LSTM, using only the RGB images and following the network and parameters available in their respective papers. The last two models are based on the two-stream EfficientNetV2-LSTM, where the first one was trained on the original full images, and the second was trained on the cropped images obtained from the dog-centered sequence spatial cropping.

Table 7 shows a comparison of dog behavior recognition performance results using each model. As shown in the table, the EfficientNet-LSTM model performed better than the other methods. In addition, the results proved the effectiveness of using dog-centered cropped images, as opposed to full images as input data, which confirms our initial assumption about the impact that the background can have on the recognition and validates the benefit of the cropping unit that was included in the system. The TDMap-CNN showed a lower F1 score compared to the other models because the method relies heavily on motion information to classify actions, and this leads to misclassifications when used with static behaviors that contain little motion. On the other hand, one-stream methods rely solely on appearance features, which affect the recognition of behaviors with similar postures. This further confirms the effectiveness of exploiting both appearance and motion features when recognizing static and active behaviors, as is the case with the proposed method. Finally, the proposed method was evaluated for the inference time required for the system to operate, including YOLOR-P6 Seq-Bbox matching object detection, sequence retrieval, spatial cropping, GMA-based optical flow extraction, and EfficientNet-LSTM inference. The total was 0.226 s/image, which confirms the capacity of the system to execute in real time.

#### 4.3.4. Dog Behavior Summarization and Visualization Results

**Figure 6:**
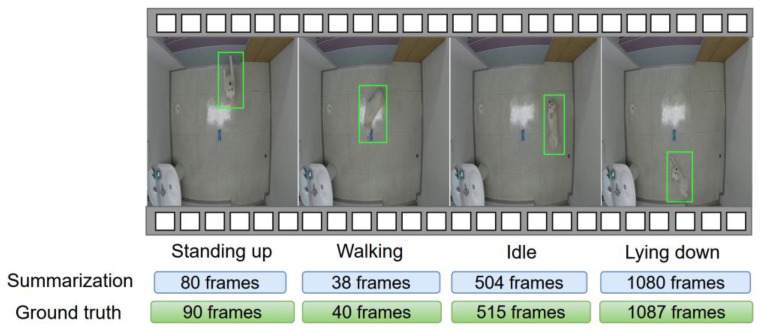
Behavior-based video summarization results on video containing different dog behaviors.

To demonstrate the effectiveness of the dog behavior-based summarization, a timeline of the recognized and summarized behaviors from a dog video is shown in Figure 6, along with the ground truth. As seen in the figure, our method delivers summarization results that are comparable with the displayed behaviors and their lengths, which further confirms its performance.

The following figures demonstrate the visual summary of the logged dog’s behaviors and movement patterns provided by the system to help the owner and experts understand and monitor the dog’s health and welfare.

**Figure 7:**
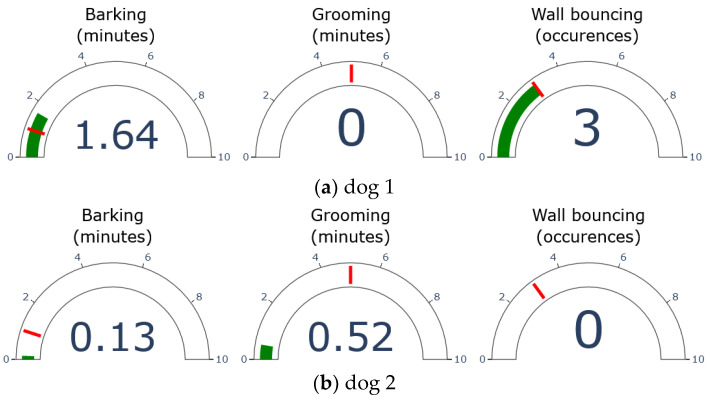
Gauge chart visualization results for monitoring of dog’s poor-welfare indicators: (**a**) example of scenario with dog 1; (**b**) example of scenario with dog 2.

Figure 7 shows an example of the real-time visualization of poor-welfare indicators using a gauge chart for each behavior during a 5 min session. The threshold is represented by the red line in each chart based on the values in Table 2. The current value for each behavior is indicated in green and displayed numerically inside each gauge. Both barking and grooming are tracked as continuous data in minutes, while wall bouncing is a discrete value for the number of times it occurred.

The charts shown in Figure 7a,b correspond to the visualization of the shih tzu’s (dog 1) and spitz’s (dog 2) poor-welfare indicators, respectively. As seen in the figures, the first dog’s wall-bouncing and barking levels both exceeded the set threshold, indicating a higher possibility of a welfare issue. Due to its past as a rescue dog, dog 1 seems to be showing signs of anxiety in this scenario. In contrast, dog 2 displayed some barking and grooming, but both were at a normal level below their respective thresholds.

**Figure 8:**
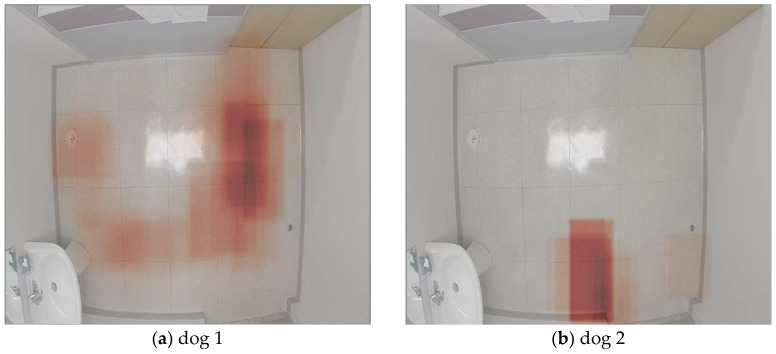
Heatmap visualization of areas where the dog spends longer time: (**a**) example of scenario with dog 1; (**b**) example of scenario with dog 2.

Figure 8 presents a summary of the dogs’ movements around the room as a heatmap to highlight the areas where they have spent significant amounts of time. Areas with a darker shade of red correspond to places where the dog spent most of its time.

In the scenario in Figure 8, both heatmaps used the tracked location data of each dog for 3.5 min right after they were left alone in the room to help assess their reaction. These first minutes are important to monitor as the period during which dogs with some health issues are likely to display abnormal behavioral patterns [78,79]. Dog 1 in Figure 8a covered a wide area of the room in a short period of time, indicating a high level of restlessness and movement, which are indicators of stress and anxiety [80,81]. Figure 8b shows how dog 2 spent that time near the exit from where its owner left, which is considered a normal behavior of attachment that healthy dogs commonly display [79].

**Figure 9:**
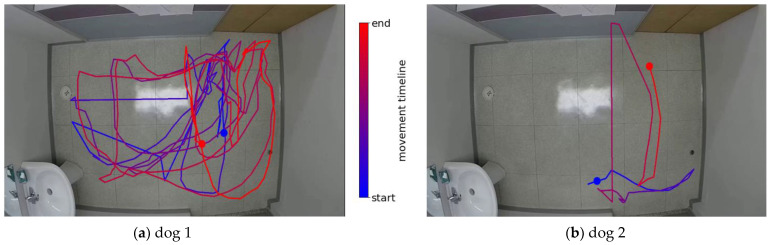
Trajectory visualization tool for displaying dog’s movement patterns: (**a**) example of scenario with dog 1; (**b**) example of scenario with dog 2.

To further detail the dog’s movement summary, the visual tool shown in Figure 9 was used to show the dog-specific trajectory.

The scenarios shown in Figure 9 represent the same ones used in Figure 8 to detail both dogs’ movement trajectories starting from the blue circle and following the color map shown in the legend and ending in the red circle. As seen in Figure 9a, dog 1 shows signs of restlessness, pacing, and a few occurrences of circling, all of which indicate a level of anxiety and possible welfare issues. In contrast, dog 2 in Figure 9b shows a simpler trajectory with a lack of exploration and activity, which suggests a relaxed state.

**Figure 10:**
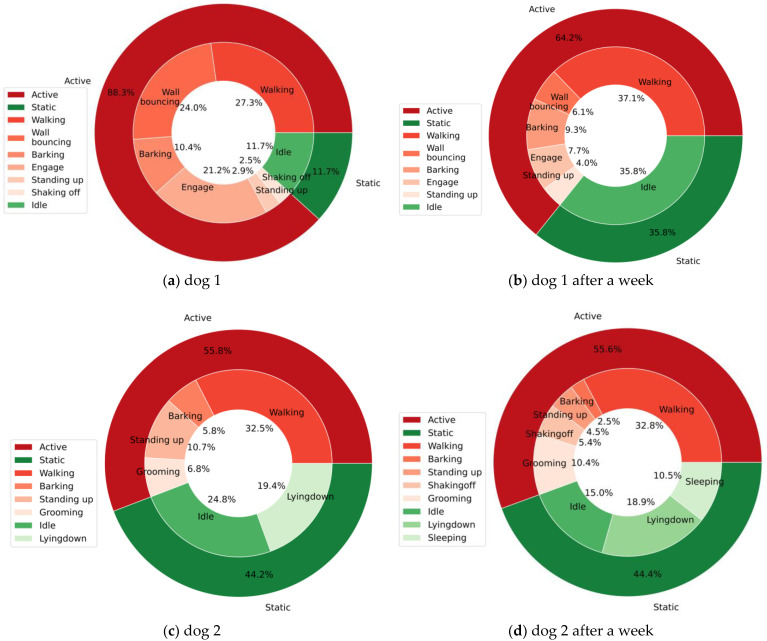
Nested doughnut charts for visual summarization of dog’s static and active behaviors: (**a**,**b**) example of two scenarios a week apart with dog 1; (**c**,**d**) example of two scenarios a week apart with dog 2.

Another visualization provided by the system is the proportion of detected dog behaviors based on logged data using a doughnut chart. The chart shows the proportion of active and static behaviors as a general category and includes a nested level detailing the proportion of specific behaviors. Examples of these visual summaries are shown in Figure 10.

Figure 10a,b both represent the proportions of the behaviors displayed by dog 1, with the first being from an early session during which the dog was still not well accustomed to the new environment, and the second representing a later session where the proportion of static behaviors relatively increased. In addition, we noticed a significant decrease in some indicators of poor welfare, such as wall bouncing, which represents a positive change and suggests that the dog’s welfare is improving. On the other hand, Figure 10c,d for dog 2 show consistency in the level of static and active behaviors displayed by dog 2 and no significant increase or decrease in activities that could indicate health and welfare issues.

**Figure 11:**
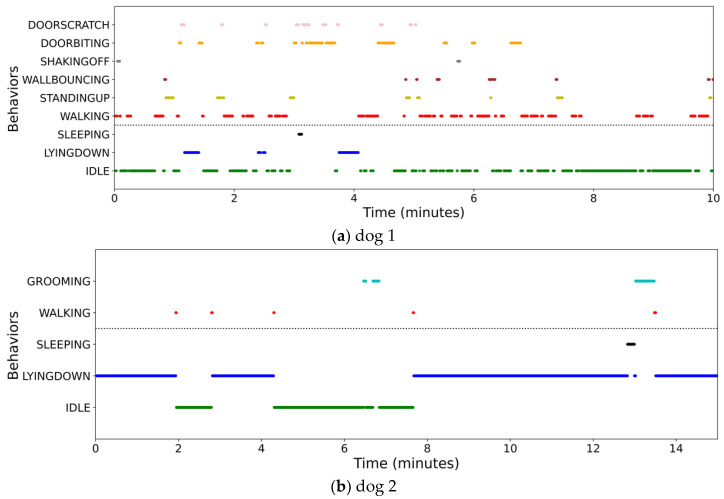
Timeline for visualization of activity and resting behaviors displayed by a dog: (**a**) example of scenario with dog 1; (**b**) example of scenario with dog 2.

Finally, Figure 11 shows the scatterplot used to draw a timeline of the dog’s behaviors to observe when and for how long behaviors occurred and to visualize the level of activity displayed. Through this timeline, it is possible to understand the dog’s behavioral patterns through time (in minutes) and monitor its level of rest and sleep, which are also important factors related to its health and welfare [75].

Figure 11a shows the timeline of dog 1 behaviors, where the static ones are listed below and separated from the active ones by a dotted line. Dog 1 in this scenario displayed a high level of activity, and although there were times when it was idle and lying down, those behaviors were scattered, and active behaviors occurred throughout the session. In addition, the dog scratched and bit the door repeatedly, indicating its attempts to escape due to lack of comfort, and did not have continuous moments of rest. In contrast, dog 2, as seen in Figure 11b, displayed a good level of comfort during the session when it spent most of its time lying down or idle, both static behaviors related to resting. Further monitoring of such rest behaviors can help owners monitor the dog’s health states because some health issues can cause an increase and decrease in resting behaviors.

#### 4.3.5. System Graphical User Interface

**Figure 12:**
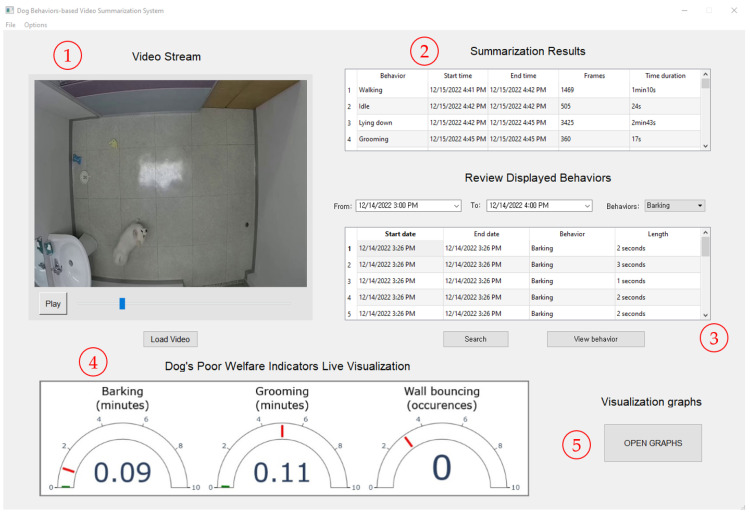
Screenshot of the main interface of the proposed system: ① controls to load and start processing a video stream; ② table displaying the behaviors summarization results; ③ controls to search for logged dog behaviors and rewatch them; ④ live display of the visualization of dog’s poor-welfare indicators; ⑤ button to open the visualization graphs interface shown in Figure 13.

**Figure 13:**
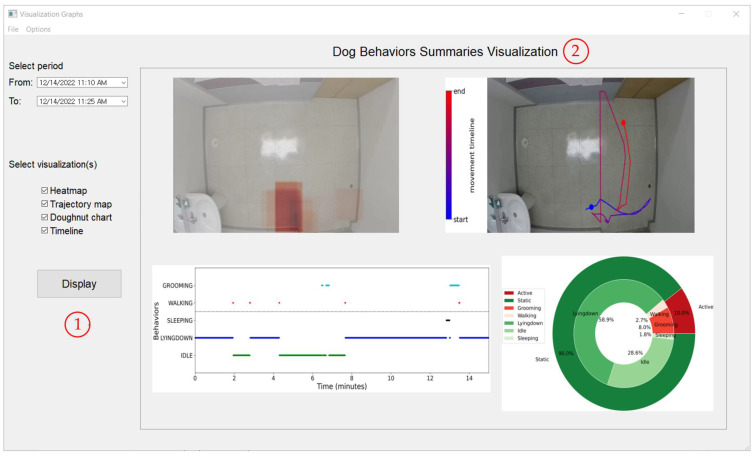
Secondary interface of the proposed system accessible through the main interface from Figure 12. ① Controls used to select the period and visualization graphs to display; ② the generated graphs of the behavior summarization to help visualize and understand the dog’s state.

A graphical user interface, shown in Figure 12 and Figure 13 below, was implemented to allow the user to easily monitor the dog’s behaviors and generate the summarization and visualization of the results.

## 5. Conclusions

A dogs’ displayed behaviors can serve as indicators that help us to understand their health and welfare, which is why it is essential for owners to monitor and keep track of them to look out for relevant signs and behavioral patterns. To provide dog owners and experts with a suitable solution, we propose a real-time system to automatically summarize dogs’ videos based on their behaviors and provide effective visual tools to help understand and analyze the dogs’ movement and behaviors. The system is composed of four consecutive modules that collect data, retrieve sequences of images where the dog is alone and spatially crops them, recognizes the dog’s behaviors, saves both them and location information as log data, and summarizes and provides visualization of the saved dog’s movement and behaviors. Dog image sequence retrieval and spatial cropping were performed using the YOLOR-P6 model followed by Seq-Bbox matching to track and save the dog’s location data, and dog-focused cropped images were then generated from each sequence to improve the behavior recognition. Subsequently, in the behavior recognition module, the GMA algorithm is used to extract the optical flow, and then both RGB and optical flows are fed as input to a two-stream EfficientNetV2-Bi-LSTM that recognizes the displayed behavior. Finally, in the last module, the behaviors are summarized and visualization tools are utilized to generate effective visual summaries of the behaviors to help owners and experts understand the dog’s health and welfare and potentially discover issues. As demonstrated through the experimental results, our system achieves an F1 score of 0.955 in terms of behavior recognition, which also proves its performance in summarization, achieving better results than other recent methods used for behavior recognition and executing in 0.23 s/image on average. Furthermore, the experiments demonstrated the effectiveness of the dog’s behavior summarization and visualization in helping owners and experts understand and monitor their health and welfare. In our future work, we intend to introduce sound data into a multimodel system to detect vocalizations such as panting and whining, as they can also provide insight into their health. In addition, we will consider introducing a system for monitoring multiple dogs, and we will explore the use of an infrared camera to monitor and recognize dog behavior under low-light conditions during the night.

## Figures and Tables

**Table 1:** Some recent studies on dog behavior recognition (published 2013–2022).

Sensor Type	Method	Real-Life Scenarios Data Containing Dog and Humans *	Real-Time Processing	Detect and Track Dog Location	Video Summarization *	Ref.
Accelerometer	Statistical classification	Not applicable	Not specified	No	Not applicable	[30]
Accelerometer	Dynamic time warping distance	Not applicable	Not specified	No	Not applicable	[31]
Accelerometer	Discriminant analysis classifier	Not applicable	Not specified	No	Not applicable	[32]
Accelerometer and gyroscope	ANN model	Not applicable	Yes	No	Not applicable	[33]
Accelerometer	FilterNet	Not applicable	Not specified	No	Not applicable	[34]
RGB camera	Faster RCNN and random forest	No	Not specified	Yes	No	[35]
Accelerometer and gyroscope	LSTM-CEP	Not applicable	Not specified	No	Not applicable	[36]
RGB camera, accelerometer and gyroscope	Multimodal CNN-LSTM	No	Not specified	No	No	[37]

* These characteristics do not apply to methods relying solely on wearable sensors, as they do not provide data that enable the verification of the presence of humans and performing video summarization. Thus, the value “not applicable” was used for such cases.

**Table 2:** Description of potentially abnormal behaviors and their thresholds.

Behavior	Description and Threshold
Barking	Excessive vocalization when dog is alone (>1 min)
Wall bouncing	Jumping and bouncing against a wall (>3 times)
Grooming	repeatedly licking own body (>5 min)

**Table 3:** Dog behavior description and dataset used for the behavior recognition model training.

Category	Behavior	Description	Data Count (Sequences of 8 Images)
Active behaviors	Barking	Vocalization usually accompanied with a slight movement of the head.	1000
	Door biting	Biting and pulling of door.	1000
	Door scratching	Scratching surface of door.	363
	Engaging toy	Making contact with and pushing around a toy with nose and mouth.	663
	Grooming	Self-licking of body parts.	1000
	Wall Bouncing	Jumping against a wall and bouncing back to the ground.	1000
	Shaking off	Twisting movement from left to right.	166
	Standing up	Standing up on back feet.	1000
	Walking	Constant movement around the room.	1000
Static behaviors	Sleeping	Lying on the side with head on the floor.	204
	Lying down	Chest, belly, and forearms in contact with the floor.	1000
	Idle	Sitting or standing on four paws with little to no motion	1000

**Table 4:** Average precision (AP), mean average precision (mAP) and average inference time of YOLOR with and without seq-bBox matching postprocessing.

	YOLOR-P6	YOLOR-P6 with seq-Bbox Matching
AP_dog_	0.903	**0.962**
AP_person_	0.863	**0.864**
mAP	0.883	**0.913**
inference time (ms)	**54.39**	54.58

**Table 5:** YOLOR-based dog sequence retrieval performance using precision, recall and F1 score.

YOLOR-Based Dog-Alone Sequence Retrieval				
Sequence	Data Count	Precision	Recall	F1 Score
Empty room	1220	0.996	0.994	0.995
Person alone	216	0.906	0.981	0.942
Dog alone	1628	0.995	0.990	0.992
Dog and Person	309	0.973	0.948	0.960
Average		0.988	0.987	0.987

**Table 6:** Proposed method behavior recognition performance using precision, recall and F1 score.

Two-Stream EfficientNetV2-LSTM (Cropped Images)				
Behavior	Data Count	Precision	Recall	F1 Score
Barking	101	0.970	0.970	0.970
Door biting	100	0.960	0.960	0.960
Door scratching	37	0.925	1.00	0.961
Engaging toy	67	0.985	1.00	0.993
Grooming	100	1.00	0.990	0.995
Idle	100	0.947	0.890	0.918
Wall bouncing	100	0.898	0.970	0.933
Lying down	101	0.952	0.990	0.971
Shaking off	18	1.00	0.944	0.971
Sleeping	21	0.955	1.00	0.977
Standing up	97	0.967	0.880	0.921
Walking	100	0.940	0.940	0.940
Average		0.956	0.956	0.955

**Table 7:** Comparison of dog behavior recognition performance using F1 score.

Behavior	F1 Score				
	TDMap-CNN	VGG16-LSTM	ResNet50-LSTM	Proposed Method (Original Images)	Proposed Method (Cropped Images)
Barking	0.782	0.881	0.926	0.929	**0.970**
Door biting	0.814	0.910	0.923	0.910	**0.960**
Door scratching	0.657	0.954	**0.985**	0.935	0.961
Engaging toy	0.735	0.942	0.983	0.992	**0.993**
Grooming	0.840	0.976	0.994	0.976	**0.995**
Idle	0.415	0.491	0.618	0.834	**0.918**
Wall bouncing	0.723	0.854	0.897	0.898	**0.933**
Lying down	0.592	0.886	0.930	0.961	**0.971**
Shaking off	0.303	0.710	0.929	0.923	**0.971**
Sleeping	0.739	0.973	0.973	**0.977**	**0.977**
Standing up	0.577	0.757	0.864	0.828	**0.921**
Walking	0.610	0.582	0.718	0.887	**0.940**
Average	0.668	0.812	0.876	0.913	**0.955**

## Data Availability

Not applicable.
